# Rapunzel Syndrome Presenting as Acute Pancreatitis With Biliary Ductal Dilatation in a Child: A Rare Mechanical Etiology of Pediatric Pancreatitis

**DOI:** 10.7759/cureus.109379

**Published:** 2026-05-21

**Authors:** Razan A Hassan, Mafaz Ombada, Yelena Korotkaya

**Affiliations:** 1 Pediatrics, University of Florida College of Medicine, Jacksonville, USA; 2 Pediatric Gastroenterology, Nemours Children's Health System, Jacksonville, USA

**Keywords:** abdominal mass, acute pancreatitis, biliary dilatation, child, gastric bezoar, intussusception, pancreatic duct, pediatric pancreatitis, rapunzel syndrome, trichobezoar

## Abstract

Rapunzel syndrome is a rare manifestation of trichobezoar in which a gastric hair mass extends beyond the pylorus into the small intestine. It typically presents with abdominal pain, nausea, vomiting, early satiety, weight loss, or bowel obstruction. Presentation of acute pancreatitis is uncommon and may delay diagnosis. We report a previously healthy nine-year-old girl who presented with progressive epigastric pain, nausea, vomiting, decreased oral intake, and constipation. Laboratory evaluation demonstrated markedly elevated lipase levels consistent with acute pancreatitis. Abdominal ultrasound revealed intrahepatic and extrahepatic biliary ductal dilatation without gallstones or choledocholithiasis. Symptoms initially improved with supportive care; however, persistent pain and a palpable epigastric mass prompted further imaging. Computed tomography demonstrated a large gastric trichobezoar extending into the jejunum, consistent with Rapunzel syndrome, with associated intussusception and compression of the pancreatic duct. The patient underwent exploratory laparotomy with complete bezoar removal, followed by resolution of symptoms and normalization of lipase levels. This case highlights Rapunzel syndrome as a rare mechanical cause of pediatric pancreatitis and emphasizes the importance of considering extrinsic compression when pancreatitis occurs with biliary dilatation and normal liver biochemical findings.

## Introduction

Trichobezoars are conglomerates of ingested hair that accumulate within the gastrointestinal tract and most commonly occur in female children and adolescents, particularly between 4 and 17 years of age [1-3]. Rapunzel syndrome is a rare and more severe variant in which the gastric trichobezoar extends beyond the pylorus into the small intestine [1,2]. Since its initial description by Vaughan et al. in 1968, fewer than 100 pediatric cases have been reported in the literature [1,2]. The syndrome is frequently associated with trichotillomania, trichophagia, psychiatric comorbidities, or behavioral disorders [2-4]. However, these classic features may be absent, potentially delaying recognition and diagnosis.

Clinical manifestations are often nonspecific and may include abdominal pain, nausea, vomiting, early satiety, weight loss, anemia, constipation, or bowel obstruction [2-5]. Because symptoms may develop gradually over time, diagnosis is frequently delayed until complications occur. Reported complications include gastric ulceration, perforation, obstruction, intussusception, obstructive jaundice, and, rarely, acute pancreatitis [2-6].

Pediatric pancreatitis is more commonly associated with biliary disease, medications, trauma, infections, systemic illness, anatomic abnormalities, or idiopathic causes. Mechanical pancreatitis caused by extrinsic compression of the pancreatic duct or ampulla by a trichobezoar is uncommon and may not be initially recognized [4-8]. Cross-sectional imaging is often necessary to establish the diagnosis, determine the extent of distal extension, and identify associated complications [5,6].

We present the case of a nine-year-old girl with Rapunzel syndrome whose initial presentation was acute pancreatitis with biliary ductal dilatation, highlighting an unusual diagnostic sequence and the importance of considering rare mechanical causes when more common etiologies are not identified.

## Case presentation

A previously healthy nine-year-old girl presented to the emergency department with one week of progressive epigastric abdominal pain, nausea, intermittent non-bilious emesis, decreased oral intake, fatigue, and constipation. Several family members had concurrent gastrointestinal symptoms, and the family initially attributed her illness to viral gastroenteritis. However, her pain persisted and progressively worsened, prompting hospital evaluation.

She localized the pain to the epigastric region with intermittent radiation to the back. There was no recent travel, toxin exposure, or history of scorpion sting. Family history was notable for maternal cholelithiasis.

**Figure 1:**
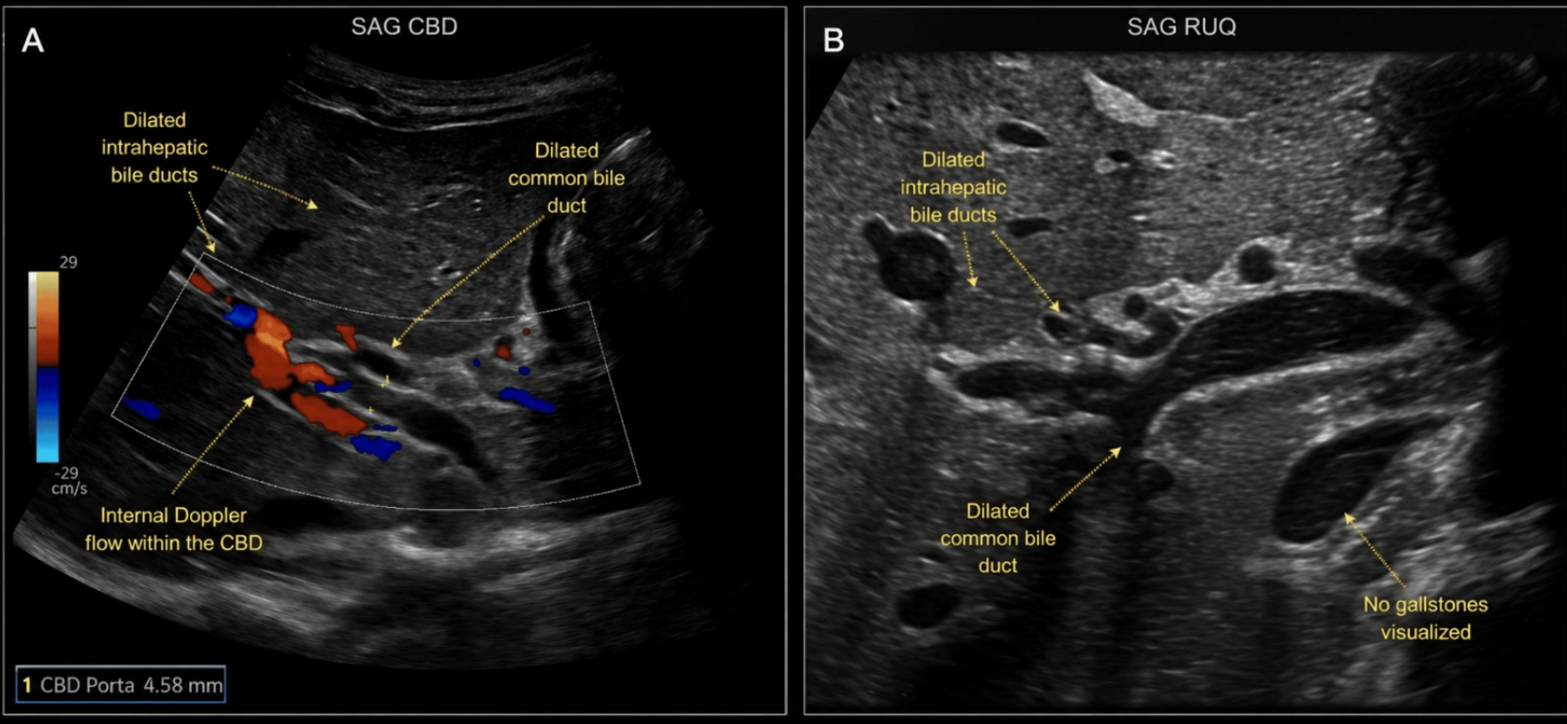
Abdominal ultrasound images demonstrating nonspecific intrahepatic and extrahepatic biliary ductal dilatation (A) Sagittal color Doppler view of the common bile duct showing mild common bile duct prominence with adjacent intrahepatic biliary ductal dilatation. (B) Sagittal grayscale right upper quadrant view demonstrating intrahepatic biliary ductal dilatation and extrahepatic duct prominence without evidence of cholelithiasis or choledocholithiasis. Trace ascites was present (not shown). Visualization of the pancreas was limited by overlying bowel gas.

Initial laboratory evaluation demonstrated a serum lipase level of 1,035 IU/L (reference range: 22-51 IU/L), consistent with acute pancreatitis. Liver transaminases and bilirubin levels were within normal limits. Pancreatitis-related laboratory trends are summarized in Table 1. Abdominal ultrasound revealed nonspecific intrahepatic and extrahepatic biliary ductal dilatation without evidence of cholelithiasis or choledocholithiasis, as shown in Figure 1. Trace ascites was present, and visualization of the pancreas was limited by overlying bowel gas.

**Table 1:** Pancreatitis-related laboratory findings Pancreatitis-related laboratory trends during the initial presentation and follow-up demonstrated markedly elevated lipase levels at presentation with normalization following surgical removal of the trichobezoar. Liver biochemical findings remained within normal limits throughout the clinical course, supporting extrinsic pancreaticobiliary compression rather than intrinsic hepatobiliary disease.

Laboratory test	Initial presentation	Follow-up	Reference range
Lipase (IU/L)	1,035 (H)	4 (L)	22-51
Total bilirubin (mg/dL)	0.3	0.2 (L)	0.3-1.8
Aspartate aminotransferase (U/L)	24	24	12-47
Alanine aminotransferase (IU/L)	10 (L)	10 (L)	14-54
Alkaline phosphatase (IU/L)	161	161	58-234

The patient was admitted for supportive management with intravenous fluids, bowel rest, and analgesia. Her symptoms improved, and she was discharged home.

At outpatient follow-up, she continued to report recurrent epigastric pain, nausea, heartburn, and early satiety. Her mother also noted a mobile abdominal mass. On examination, the patient appeared thin but was in no acute distress. The abdomen was soft and non-distended with normal bowel sounds, and a palpable epigastric mass was appreciated without guarding or rebound tenderness. Scalp examination demonstrated normal hair distribution without alopecia, thinning, or patchy hair loss.

**Figure 2:**
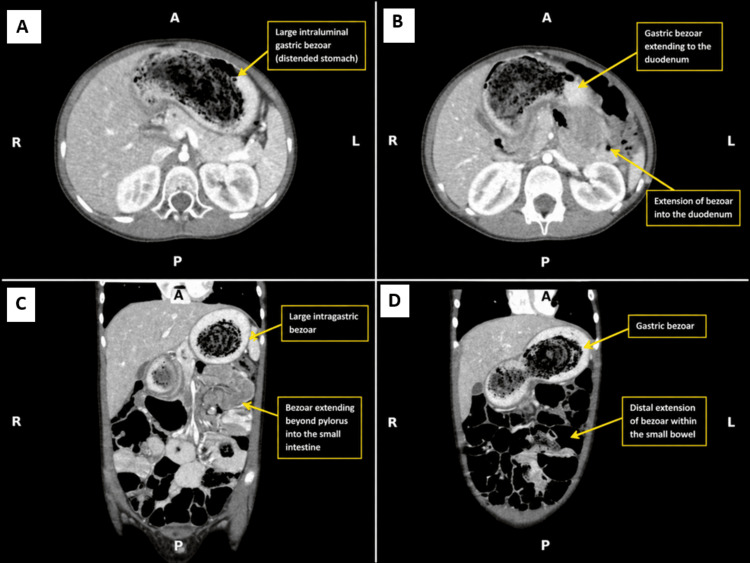
Contrast-enhanced CT of the abdomen demonstrating Rapunzel syndrome with gastric trichobezoar extension into the jejunum, intussusception, and pancreatic compression (A, B) Axial contrast-enhanced CT images demonstrate a large mottled intraluminal gastric trichobezoar occupying the stomach with extension beyond the pylorus. (C, D) Coronal reformatted images demonstrate the elongated bezoar tail extending into the proximal jejunum, consistent with Rapunzel syndrome, with associated small bowel telescoping/intussusception and mass effect on the pancreatic region. Orientation markers: A = anterior, P = posterior, R = right, and L = left.

Given the persistent symptoms and concerning examination findings, computed tomography of the abdomen and pelvis with contrast was obtained. Imaging demonstrated a large heterogeneous intraluminal gastric mass with a characteristic mottled appearance caused by entrapped air and debris, consistent with a gastric trichobezoar. The bezoar extended beyond the pylorus with an elongated tail into the jejunum, measuring approximately 40 cm, confirming Rapunzel syndrome, as illustrated in Figure 2. Associated findings included jejunojejunal intussusception, mild pancreatic ductal dilatation, and compression of the pancreatic head and neck.

The patient subsequently underwent exploratory laparotomy with gastrotomy and enterotomy for complete bezoar removal. Postoperatively, she had an uncomplicated recovery with gradual advancement of diet and return of bowel function. Her abdominal pain resolved, and serum lipase levels normalized following surgical intervention, as summarized in Table 1. She was discharged home in stable condition with recommendations for outpatient follow-up.

## Discussion

Rapunzel syndrome is a rare and severe variant of trichobezoar in which a gastric hair mass extends through the pylorus into the small intestine. Since its first description, most reported cases have involved children or adolescent females and are frequently associated with trichotillomania, trichophagia, psychiatric comorbidities, or visible alopecia [1,2]. However, these classic features are not universally present. Our patient had no known psychiatric history or scalp alopecia, illustrating that the absence of external clues should not exclude the diagnosis.

Most pediatric cases present with chronic abdominal pain, nausea, vomiting, early satiety, weight loss, anemia, or symptoms of gastric outlet or small bowel obstruction [2-4]. In a retrospective pediatric review by Liang et al., common presenting features included abdominal pain, vomiting, palpable abdominal mass, and nutritional compromise, while acute pancreatitis with cholecystitis was reported in only a minority of patients [4]. This case is therefore notable because acute pancreatitis preceded the diagnosis of trichobezoar, an uncommon presentation that may delay recognition.

The presumed mechanism of pancreatitis in this setting is mechanical obstruction caused by mass effect at the level of the duodenum, ampulla of Vater, or pancreatic duct outflow tract. Extension of a trichobezoar into the duodenum can result in compression of the pancreatic head or ampulla, leading to impaired pancreatic outflow and pancreatitis, a phenomenon described in rare case reports [7,8]. In our patient, intrahepatic and extrahepatic biliary ductal dilatation occurred without gallstones, choledocholithiasis, or abnormal liver biochemical findings, supporting extrinsic compression rather than intrinsic biliary disease. Resolution of symptoms and normalization of lipase levels following bezoar removal further support a causal relationship. Mirza et al., in a series of 17 gastrointestinal trichobezoar cases, reported only one patient with transient pancreatitis, emphasizing the rarity of this complication [5].

Diagnosis may be challenging because abdominal ultrasound, although commonly used as first-line imaging in children, may fail to identify bezoars because of overlying bowel gas or limited gastric visualization. Cross-sectional imaging with CT is highly valuable because it can establish the diagnosis, define distal extension, and identify complications such as obstruction or intussusception [6]. CT findings typically demonstrate a well-defined intraluminal mass with a characteristic mottled appearance due to entrapped air and debris [6,9]. In our case, persistent symptoms after discharge and the finding of a palpable epigastric mass prompted CT imaging, which confirmed Rapunzel syndrome with jejunojejunal intussusception.

Management depends on the bezoar size and extent. While small gastric bezoars may occasionally be removed endoscopically, Rapunzel syndrome generally requires surgery because of distal extension and the risk of obstruction, ischemia, perforation, or recurrence [2,3]. Operative extraction remains the definitive treatment, followed by psychiatric or behavioral evaluation to reduce recurrence risk. Multidisciplinary care, including psychological assessment, is essential given the risk of recurrence and underlying behavioral contributors [7].

This case highlights an important clinical pearl: unexplained pancreatitis with biliary ductal dilatation and normal liver enzyme levels, particularly when followed by persistent epigastric pain, early satiety, or a palpable abdominal mass, should prompt consideration of rare mechanical causes such as Rapunzel syndrome.

## Conclusions

Rapunzel syndrome is a rare but important mechanical cause of pediatric pancreatitis and biliary ductal dilatation. This case demonstrates that acute pancreatitis may precede the diagnosis of trichobezoar, even in the absence of alopecia, known trichophagia, or a psychiatric history. Persistent epigastric pain, early satiety, or a palpable abdominal mass following apparent resolution of pancreatitis should prompt further evaluation with cross-sectional imaging.

Early recognition is essential to prevent complications such as obstruction, intussusception, ischemia, or recurrent pancreatitis, and definitive management typically requires surgical removal with consideration of behavioral or psychiatric follow-up to reduce the risk of recurrence.
